# PPAR-*γ* Agonist Alleviates Liver and Spleen Pathology via Inducing Treg Cells during *Schistosoma japonicum* Infection

**DOI:** 10.1155/2018/6398078

**Published:** 2018-07-17

**Authors:** Yuxiao Zhu, Yangyue Ni, Ran Liu, Min Hou, Bingya Yang, Jingwei Song, Hongzhi Sun, Zhipeng Xu, Minjun Ji

**Affiliations:** ^1^The Affiliated Sir Run Run Hospital of Nanjing Medical University, Nanjing, Jiangsu 211100, China; ^2^Department of Pathogen Biology, Nanjing Medical University, Nanjing, Jiangsu 211166, China; ^3^Jiangsu Province Key Laboratory of Modern Pathogen Biology, Nanjing, Jiangsu 211166, China

## Abstract

**Background:**

Peroxisome proliferator-activated receptor- (PPAR-) *γ* plays critical roles in human metabolic disorders and has recently been implicated as a regulator of cellular proliferation and inflammatory responses. Regulatory T cells (Tregs), which express high levels of PPAR-*γ* protein, have the ability to maintain immune tolerance to self-antigens and regulate immune response to *Schistosoma* infection. However, mechanisms involved in the resolution of these responses are elusive.

**Methods:**

Liver and spleen tissue samples in *Schistosoma japonicum*-infected mice after administration of pioglitazone (a PPAR-*γ* agonist) were collected. The hepatic and splenic pathologies were detected by H&E and Masson staining. The percentages of Th1/2 and Treg cells in the liver and spleen of each mouse were determined using flow cytometry. Levels of gene expression of PPAR-*γ* and Foxp3 in tissues or cells were determined using real-time PCR (RT-PCR). Macrophages were treated with pioglitazone *in vitro* or cocultured with normal purified CD4^+^ T cells for detecting Treg cells by flow cytometry. The interactions of PPAR-*γ* with Foxp3 in CD4^+^ T cells were detected by coimmunoprecipitation.

**Results:**

Administration of pioglitazone resulted in the prevention of the development of hepatic and splenic pathologies. Activation of PPAR-*γ* by pioglitazone resulted in increased percentages of CD4^+^CD25^+^Foxp3^+^ Treg cells and decreased percentages of CD3^+^CD4^+^IFN-*γ*^+^ and CD3^+^CD4^+^IL-4^+^ cells in the liver and spleen of *Schistosoma japonicum*-infected mice. In addition, the PPAR-*γ* agonist can induce Treg cells *in vitro* directly or by modulating the macrophage's function indirectly. Furthermore, through interaction with Foxp3 in CD4^+^ T cells, the PPAR-*γ* agonist can promote the expression of Foxp3; however, the inhibitor of PPAR-*γ* weakened the expression of Foxp3 by modifying the coexpression of Foxp3 and PPAR-*γ*.

**Conclusions:**

Our study reveals a previously unrecognized role for PPAR-*γ*/Foxp3 signaling in regulating the immunopathology that occurs during *Schistosoma* infection through induction of Treg cells.

## 1. Introduction

Schistosomiasis, caused by schistosomes, remains a major public health problem in many countries in Asia and Europe [1, 2]. The most serious schistosomiasis immune pathogenesis is the hepatic granuloma formation around deposited eggs and subsequent fibrosis, which are orchestrated by the CD4^+^ T cell response [3, 4]. During infection, an initial proinflammatory Th1-type polarized response is continuously triggered by schistosome-soluble adult worm antigen fractions, with elevated interferon-*γ* (IFN-*γ*), tumor necrosis factor-*α* (TNF-*α*), and interleukin-12 (IL-12) levels. After a large number of deposited schistosome eggs, the process of granuloma formation in the liver is triggered, which undergoes an obvious shifting from an initial restrictive Th1-type response to a late Th2-dominant response [5]. Importantly, schistosome infections are renowned for their ability to induce powerful regulatory networks including regulatory T cells that regulate schistosome egg-induced pathology [5–7]. However, the mechanism underlying regulatory cell-controlled liver pathology is still largely unknown.

Peroxisome proliferator-activated receptor- (PPAR-) *γ*, an important fatty acid-activated nuclear receptor, plays a critical role in human metabolic disorders, such as dyslipidemia and insulin resistance [8, 9]. PPAR-*γ* also plays an important role in the regulation of immune response-related fibrosis. One of the PPAR-*γ* agonists (rosiglitazone) has been reported to prevent murine hepatic fibrosis, which was accompanied by the induction of the expression of TNF-*α* and IL-6 but a reduction of the expression of TGF-*β*1 and *α*-SMA in liver tissue [10]. In the CCL4-induced rat liver fibrosis model, infection with the recombinant lentiviral expression vector carrying the rat PPAR-*γ* gene resulted in suppressing hepatic stellate cell (HSC) proliferation and hepatic fibrosis and inhibiting the expression of *α*-SMA and type I collagen [11]. However, the potential mechanisms of the PPAR-*γ* agonist on the development of *Schistosoma* egg-induced liver pathology are still not fully understood.

Evidence suggested that PPAR-*γ* is a crucial transcription factor for regulating Treg cell accumulation and function, and the specific ablation of PPAR-*γ* in Treg cells greatly reduced the population of Treg cells accumulated in visceral adipose tissue (VAT) [12]. PPAR-*γ* agonists (ciglitazone and 15-deoxy-Δ-^12,14^-PG J2) as molecules could significantly increase Foxp3 expression in human iTregs [13]. PPAR-*γ* agonist- (pioglitazone) attenuated upper airway allergic inflammation may be mediated by the induction of Tregs [14]. In addition, our previous data showed that pioglitazone could increase regulatory T cells in the VAT of high-fat-diet mice [15]. In view of the importance of PPAR-*γ* in the regulation of immune response, we attempt to examine not only the effectiveness of pioglitazone but also shed light on the significance of the PPAR-*γ*/Foxp3 axis to regulate schistosome egg-induced liver pathology during *S. japonicum* infection.

## 2. Materials and Methods

### 2.1. Ethics Statement, Animal, Parasites and Antigen Preparation

Six-week-old C57BL/6 mice were obtained from the Model Animal Research Center of Nanjing University and kept in specific pathogen-free environments in the Animal Care Facility of Nanjing Medical University. All experiments were performed in strict accordance with the Regulations for the Administration of Affairs Concerning Experimental Animals (1988-11-01). All of the animal experiments were approved by the Nanjing Medical University Animal Ethics Committee (number 1601004).

Cercariae were collected from *S. japonicum*-infected snails, which were bought from the Jiangsu Institute of Parasitic Diseases (Wuxi, China).

Soluble egg antigen (SEA) was prepared carefully to prevent endotoxin contamination. The concentration of SEA was measured using the Bicinchoninic Acid Protein Assay Kit (Pierce, USA). The concentration of endotoxin in SEA was below 0.03 EU/ml using a timed gel endotoxin detection kit (Sigma-Aldrich, USA).

### 2.2. Experimental Mice Model

Mice were divided into four groups randomly as follows: group I was identified as normal control (uninfection) and group II was treated with pioglitazone only (uninfection + PIO). Group III was infected with 10 ± 1 *S. japonicum* cercariae through the skin (infection), and group IV was treated with pioglitazone 4 weeks post *S. japonicum* infection (infection + PIO). Pioglitazone (10 mg/kg) was given by intragastric means every other day for 5 weeks in groups of uninfection + PIO and infection + PIO. Meanwhile, mice in the uninfection group and infection group were given normal saline for 5 weeks. All mice were sacrificed at 9 weeks post infection.

### 2.3. Histopathological Examination

The sections of livers were examined with hematoxylin and eosin (H&E) staining and Masson staining. The sectioned liver tissue was fixed in 4% paraformaldehyde, embedded in paraffin and stained according to standard protocols. Single-egg granulomas were examined and sizes were calculated using AxioVision Rel 4.7 (Carl Zeiss GmbH, Jena, Germany). Additionally, the degree of hepatic fibrosis was evaluated using a professional image analysis software (Image Pro Plus).

Eggs in the liver were calculated after putting 0.2 mg of liver in 10% KOH overnight and counting the number of eggs by taking 10 *μ*l three times.

Hepatic mass index (HMI) was calculated by the liver weight and body weight. The same was done with the spleen index.

### 2.4. Cell Preparation and Stimulation

Spleen lymphocytes were prepared as in a previous study [16]. Briefly, single cell suspensions were prepared by collecting the cells after the spleen was ground in incomplete RPMI 1 × 1640 medium (Gibco, USA), followed by red blood cell (RBC) lysis, washing by staining buffer which contained PBS with 1% FBS, and then filtering through a 200 gauge mesh.

Hepatic lymphocytes were prepared as described before [16]. Briefly, mouse livers were perfused and then the excised liver was cut into small pieces and incubated in digestion buffer (collagenase IV/dispase mix, Invitrogen, Carlsbad, CA). The digested liver tissue was then homogenized using a MediMachine with 50 mm Medicons (Becton Dickinson, San Jose, CA) for 5 min at low speed. The liver suspension was then centrifuged at low speed to sediment the hepatocytes. The remaining cells were separated on a 35% Percoll (Sigma-Aldrich) gradient by centrifuging at 600 ×g. The lymphocyte fraction was resuspended in red cell lysis buffer and then washed in 10 ml of complete RPMI 1640.

For CD4^+^ T cell preparation of the spleen, purified CD4^+^ T cells were acquired by MACS (magnetic-activated cell sorting) according to the manufacturer's protocols (Miltenyi Biotec). CD4^+^ T cells were then stimulated with PIO (10 *μ*g/ml), PIO + GW9662 (a potent antagonist of PPAR-*γ*) (10 *μ*g/ml + 2 ng/ml), SEA (25 *μ*g/ml), SEA + GW9662 (25 *μ*g/ml + 2 ng/ml), and PIO + SEA (10 *μ*g/ml + 25 *μ*g/ml) for 24 h.

For peritoneal macrophage preparation, peritoneal exudate cells (PECs) from each mouse were harvested using lavage with ice-cold PBS with 1% FBS. PECs were resuspended in RPMI medium containing 10% FBS and 1% penicillin streptomycin (PS). Nonadherent cells were removed after 2 hours of incubation. Adherent macrophages were incubated with 5 mM EDTA/PBS for 10 min at 37°C, and the cell purity marked with F4/80 was detected by flow cytometry (~98%) [16]. Then, peritoneal macrophages were treated with the above stimulants for 24 h. After that, macrophages were scoured off and cultured with purified CD4^+^ T cells for 24 h, and the cells were collected for Treg cell analysis by flow cytometry.

### 2.5. Flow Cytometry

For Th1/Th2 analysis, 2 × 10^6^ cells per ml were stimulated for 6 h at 37°C in 5% CO_2_ with 0.7 *μ*l of GolgiPlug (BD Biosciences, USA), 25 ng/ml of PMA (Sigma-Aldrich), and 1 *μ*g/ml of ionomycin (Sigma-Aldrich). After the staining of surface markers (CD3-APC, CD4-FITC), cells were then washed with staining buffer and fixed and permeabilized with the Fix&Perm Cell Permeabilization Kit (Medium A/Medium B) (Life Technologies) and then stained with antibodies against INF-*γ*-PE or IL-4-PE. Finally, cells were washed twice and resuspended in staining buffer prior to flow cytometry analysis.

For Treg analysis, 2 × 10^6^ cells were stained with CD4-FITC and CD25-PE-CY7 for 30 minutes, and then cells were washed with staining buffer and fixed and permeabilized with the Fix&Perm Cell Permeabilization Kit (Life Technologies) according to the manufacturer's instructions. Cells were incubated for 40 min at 4°C in the dark. Subsequently, cells were blocked by Fc and then stained with Foxp3-PE. Finally, cells were washed twice and resuspended in staining buffer prior to flow cytometry analysis.

### 2.6. Real-Time PCR Analysis

For real-time PCR analysis, total RNA was extracted using the Total RNeasy kit (TaKaRa, Tokyo) and cDNA was prepared using random hexamer primers (GeneWiz, Grand Island). Primer sequences used were as follows: Foxp3 [17], forward: GGCCCTTCT CCAGGACAGA, reverse: GCTGATCATGGCTGGGTTGT; PPAR-*γ*, forward: GCGGCTGAGAAATCACGTTC, reverse: GAATATCAGTGGTTCACCGCTTC; and GADPH, forward: AACTTTGGCATTGTGGAAGG, reverse: GGATGCAGGGATGATGTTCT. Gene expression was quantified using the LightCycler® 96 System (Roche Life Science, Switzerland). Amplification was performed in a total volume of 20 *μ*l for 40 cycles and products were detected using SYBR Green I dye (Roche Life Science, Switzerland). The relative expression of mRNA was calculated using a comparative method (2^−ΔΔct^) according to the ABI Relative Quantification Method.

### 2.7. Coimmunoprecipitation

Coimmunoprecipitation was performed for the determination of the interaction between Foxp3 and PPAR-*γ* in CD4^+^ T cells. Briefly, the lysates (~200 *μ*g of protein) of purified CD4^+^ T cells were incubated with rocking at 4°C with anti-Foxp3 (rabbit anti-mouse; cat: 222298-1-AP used at 1 : 500 dilution, Proteintech) or anti-PPAR-*γ* (rabbit anti-mouse; cat: 16643-1-AP used at 1 : 500 dilution, Proteintech). After 2 h, the immune complexes were incubated with protein A/G-plus agarose beads (Santa Cruz Biotechnology, Santa Cruz, CA) overnight at 4°C. The immunopurified proteins were washed and immunoblotted using specific antibodies.

### 2.8. Statistical Analyses

All analyses were carried out with the SPSS 21.0 software. Data were shown as mean ± SD. Multiple comparisons were performed by one-way ANOVA, and followed by LSD posttest for comparison between two groups. Significance was considered when *P* values < 0.05. We used GraphPad Prism 5.0 software (GraphPad Software Inc., La Jolla, CA, USA) for all graphical representations.

## 3. Results

### 3.1. PPAR-*γ* Agonist Alleviates Hepatic and Splenic Pathology

**Figure 1:**
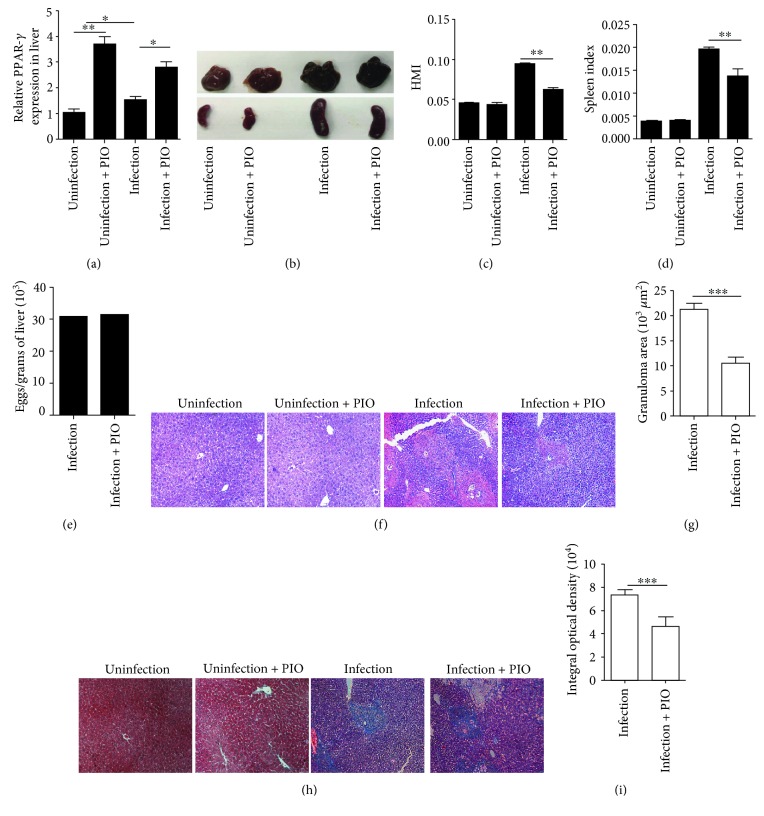
The role of the PPAR-*γ* agonist in hepatic and splenic pathologies. (a) The mRNA level of PPAR-*γ* in the liver of mice in each group after continuous intragastric administration with pioglitazone for 5 weeks. Data are expressed as the mean ± SD of 6 mice for each group. (b–d) Hepatic mass index (HMI) and spleen index were calculated by the ratio of the liver or the spleen weight to the body weight, respectively. (e) The numbers of eggs extracted from the liver (1 gram) in each mice were determined by microscopic examination. (f, h) Paraffin-embedded liver sections stained with H&E or Masson; original magnification, ×100. (g) For each mouse, the sizes of 20 liver granulomas around single eggs were quantified with AxioVision Rel 4.7. Paraffin-embedded sections were stained with Masson; original magnification, ×100; (i) the mean optical density of collagen fibers by Masson staining was digitized and analyzed on Image Pro Plus software. Data are expressed as the mean ± SD of 12 mice for each group, which resulted from two independent experiments. ^∗^*P* < 0.05, ^∗∗^*P* < 0.01, and ^∗∗∗^*P* < 0.001 (ANOVA/LSD).

To investigate the impact of pharmacological modulation of pioglitazone on hepatic pathology, we first detected the expression of PPAR-*γ* in the liver after five weeks post treatment. Results showed that pioglitazone treatment could significantly increase hepatic PPAR-*γ* expression (*P* < 0.001) (Figure 1(a)). Additionally, we weighed the liver, spleen, and body weight of each mouse and calculated the hepatic mass index (HMI) and spleen index. Results showed that the HMI and spleen index decline when infected mice received pioglitazone treatment (*P* < 0.001; *P* < 0.001) (Figures 1(b)–1(d)). We also calculated the number of eggs in the liver. Results showed that pioglitazone had little impact on egg production (Figure 1(e)). In addition, H&E and Masson staining results showed that treatment with pioglitazone significantly reduced the average area of single-egg granulomas (Figures 1(f) and 1(g)) and ameliorated liver fibrosis (Figures 1(h) and 1(i)). Together, these data showed that pioglitazone played an important role in moderating hepatic and splenic pathologies during *S. japonicum* infection.

### 3.2. PPAR-*γ* Agonist Increases the Treg Cells and Inhibits Effector Cell Response

**Figure 2:**
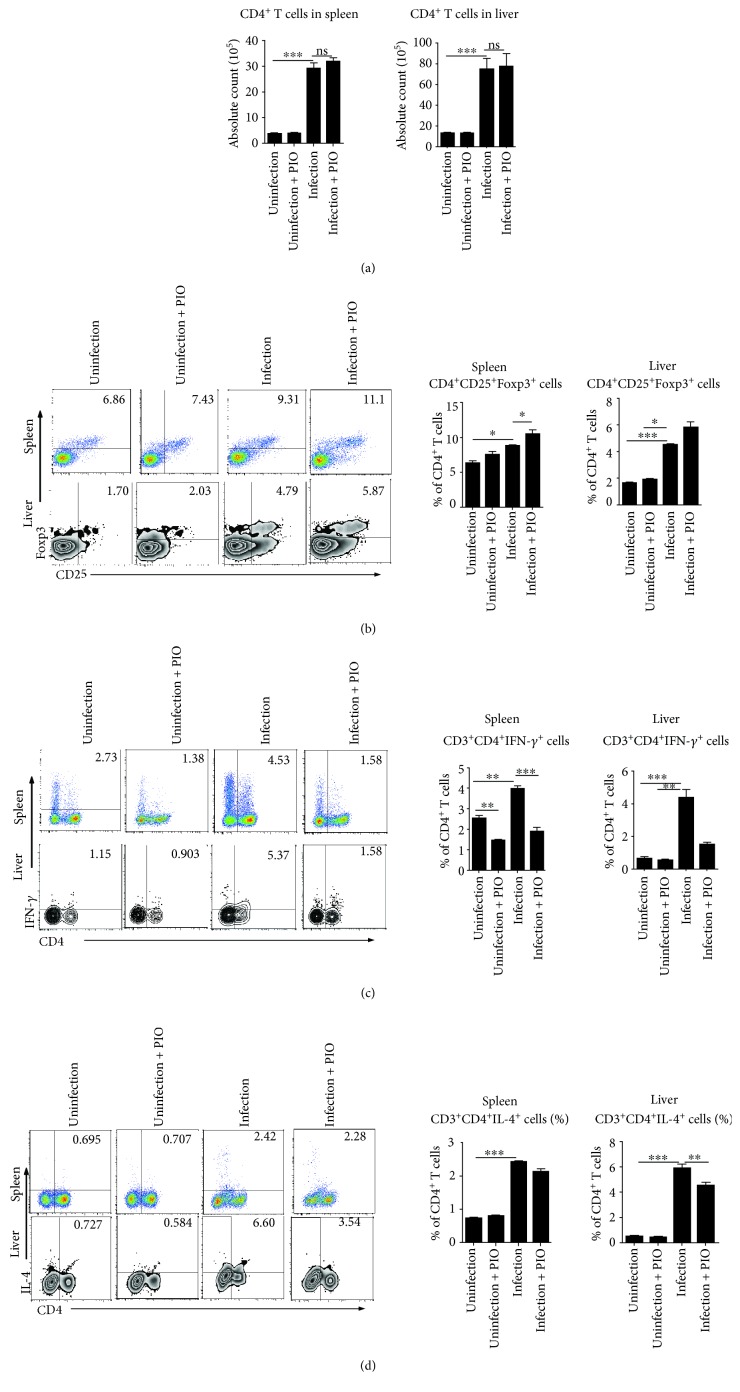
The expression of regulatory cells and effector Th1/Th2 cells after pioglitazone treatment of *S. japonicum* infection. (a–d) Single cell suspensions of mouse spleen or liver from pioglitazone-treated mice infected with or without *S. japonicum* were prepared. (a) The absolute numbers of CD4^+^ T cells both in the liver and spleen of mice were calculated as the proportion of CD3^+^CD4^+^ T cells in the leukocyte gate multiplied by the total cell count. (b) Cells were stained with CD25-APC and CD4-FITC and then intracellularly stained with PE-conjugated antibodies against Foxp3 for FACS analysis of CD4^+^CD25^+^Foxp3^+^ (Treg). (c, d) Cells were stained with CD3-APC and CD4-FITC and then intracellularly stained with PE-conjugated antibodies against IFN-*γ* or IL-4 for FACS analysis of CD3^+^CD4^+^IFN-*γ*^+^ (Th1) or CD3^+^CD4^+^IL-4^+^ (Th2) cells, respectively. Data are expressed as the mean ± SD of 12 mice for each group from three independent experiments (ANOVA/LSD), ^∗^*P* < 0.05, ^∗∗^*P* < 0.01, and ^∗∗∗^*P* < 0.001.

As CD4^+^ T cells orchestrate the development of immunopathology in schistosomiasis [5], we first investigated whether CD4^+^ T cells were regulated after pioglitazone treatment during *S. japonicum* infection (Figure 2(a)). Our results showed that the absolute numbers of CD4^+^ T cells both in the liver and spleen of pioglitazone-treated mice were not changed after *S. japonicum* infection. Previous studies showed that regulatory T cells play critical roles in regulating the development of immunopathology in schistosomiasis [18, 19], our results showed that the proportion of Treg cells both in the liver and spleen of pioglitazone-treated mice was apparently increased after pioglitazone treatment in *S. japonicum*-infected mice when compared with untreated infected mice (*P* < 0.001; *P* < 0.001) (Figure 2(b)). Altogether, these results suggested that pioglitazone may participate in regulating immunopathology through inducing Treg cells after *S. japonicum* infection.

Since Treg cells play important roles in suppressing both Th1 and Th2 responses during *Schistosoma* infection [20, 21], we next examined whether Th1 and Th2 cells were regulated by pioglitazone treatment during *S. japonicum* infection. Results showed that Th1 response (CD3^+^CD4^+^IFN-*γ*^+^) was significantly decreased both in the liver and spleen of pioglitazone-treated mice post *S. japonicum* infection (*P* < 0.001; *P* < 0.001) (Figure 2(c)). In addition, our data also showed that Th2 (CD3^+^CD4^+^IL-4^+^) cells were significantly reduced in the liver but only slightly decreased in the spleen of pioglitazone-treated mice when compared with untreated infected mice (*P* < 0.001) (Figure 2(d)).

### 3.3. PPAR-*γ* Agonist Induces Foxp3 and Augmented Treg Cells *In Vitro*

**Figure 3:**
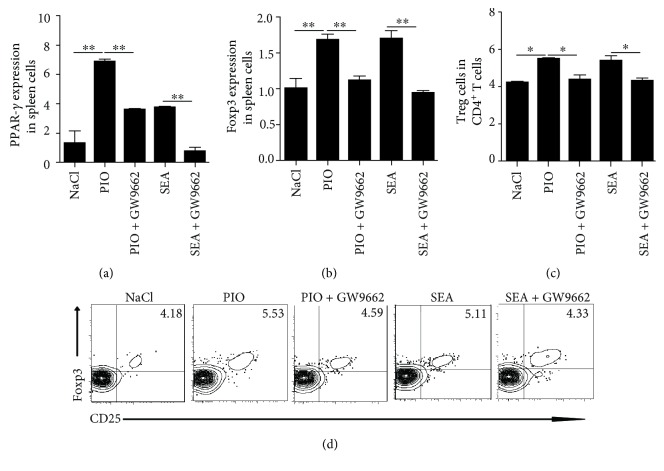
The expression of Foxp3 and Treg cells after pioglitazone or (and) SEA stimulation *in vitro.* (a, b) Splenocytes from WT mice were prepared and stimulated by pioglitazone (PIO, 10 *μ*g/ml) and a PPAR-*γ* antagonist (GW9662, 2 ng/ml) with or without SEA (25 *μ*g/ml) stimulation for 24 h, and then the mRNA level of PPAR-*γ* and Foxp3 were analyzed by RT-PCR. (c, d) The percentage of Treg was detected after pioglitazone treatment in spleen cells with or without SEA stimulation for 24 h. All experiments were repeated three times with similar results (ANOVA/LSD). ^∗^*P* < 0.05 and ^∗∗^*P* < 0.01.

Given that PPAR-*γ* is a crucial transcription factor for promoting Treg cell expression and accumulation [22], we used pioglitazone and GW9662 (PPAR-*γ* antagonist) to stimulate splenic mononuclear lymphocytes under SEA stimulation *in vitro*, and detected the expression of Foxp3, an important transcription factor of Treg cells. Results showed that treatment with the PPAR-*γ* agonist or SEA induced the mRNA levels of Foxp3 in the spleen cells; however, the expression of Foxp3 was decreased after PPAR-*γ* antagonist treatment (Figures 3(a) and 3(b)). In addition, we also found that the percentage of Treg cells was increased after PPAR-*γ* agonist or (and) SEA treatment, while PPAR-*γ* antagonist blocked this enhancement (Figures 3(c) and 3(d)). Taken together, these data suggested that the activation of PPAR-*γ* signaling could promote the production of CD4^+^CD25^+^Foxp3^+^ Treg cells.

### 3.4. PPAR-*γ* Agonist Promotes Treg Cell Differentiation through Activating Macrophages

**Figure 4:**
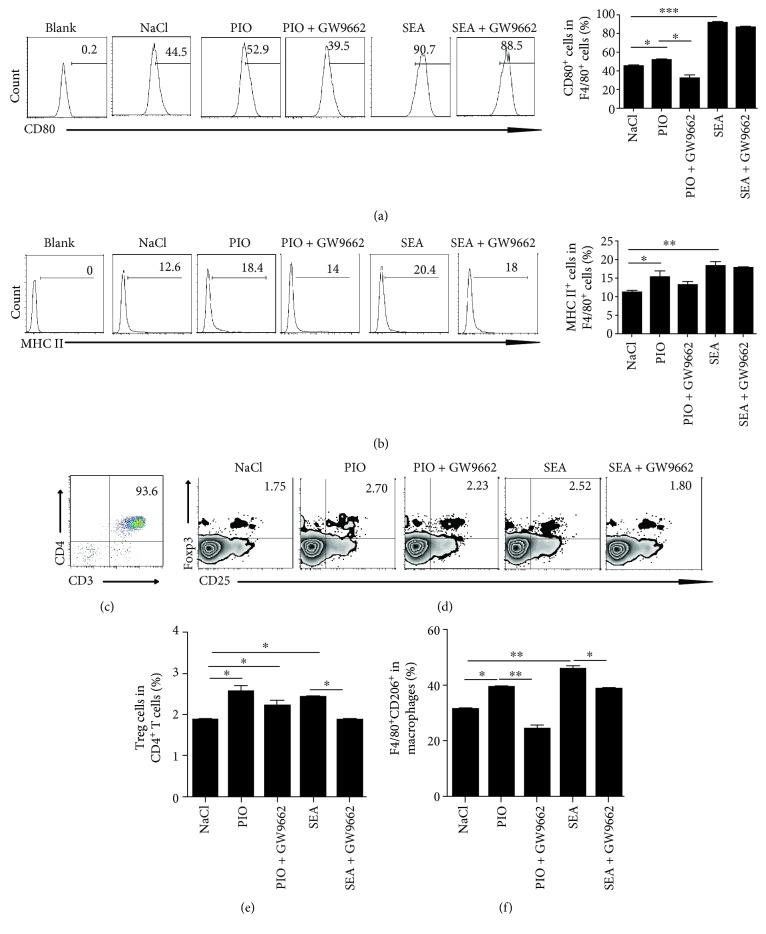
PPAR-*γ* signaling promotes Treg differentiation involved in macrophages. (a, b) Peritoneal macrophages (1 × 10^6^) were treated with pioglitazone (10 *μ*g/ml) with or without SEA (25 *μ*g/ml). The expression of CD80^+^F4/80^+^ or MHCII^+^F4/80^+^ was evaluated by flow analysis. (c) CD4^+^ (CD3^+^CD4^+^) T cells from normal mice were prepared by magnetic-activated cell sorting. Flow analysis results show that purity was ~94%. (d, e) Macrophages were treated with pioglitazone (10 *μ*g/ml) with or without SEA (25 *μ*g/ml) and cocultured with purified CD4^+^ T cells for 24 h, then Treg (CD4^+^CD25^+^Foxp3^+^) cells were analyzed by FACS. (f) After peritoneal macrophages (1 × 10^6^) were treated with pioglitazone (10 *μ*g/ml) with or without SEA (25 *μ*g/ml), the percentage of M2 macrophages (CD206^+^F4/80^+^) was evaluated by flow analysis. All experiments were repeated three times with similar results (ANOVA/LSD), ^∗^*P* < 0.05 and ^∗∗^*P* < 0.01.

It is given that macrophages serve as professional antigen-presenting cells (APCs) and play a key role in regulating adaptive immune responses [23]. To investigate whether PPAR-*γ* signaling in macrophages could affect the differentiation of CD4^+^ T cells, we firstly treated macrophages with pioglitazone with or without SEA, and analyzed the activation of macrophages. Results showed that pioglitazone-stimulated macrophages significantly increased the expression of CD80 or MHCII in macrophages (Figures 4(a) and 4(b)). In addition, the activated macrophages by pioglitazone or SEA were then cocultured with normal purified CD4^+^ T cells (Figure 4(c)). Results showed that pioglitazone pretreated macrophages significantly promoted the percentage of Treg cells with or without SEA stimulation; however, PPAR-*γ* inhibitor-pretreated macrophages could inhibit the proportion of Treg cells (*P* < 0.001) (Figures 4(c)–4(e)).

As PPAR-*γ* can be expressed in alternatively activated (M2) macrophages [24, 25], which are the main cellular constituents of granulomas [5] and important regulators in schistosomiasis [26, 27], we investigated whether PPAR-*γ* signaling regulates the M2 macrophage polarization. Results showed that pioglitazone stimulation increased CD206^+^ macrophages; however, the PPAR-*γ* antagonist dampened the expression of CD206^+^ macrophages (Figure 4(d)). Altogether, these results suggested that pioglitazone promotes CD4^+^ T cell differentiation into Treg cells through the interaction with macrophages.

### 3.5. PPAR-*γ* Promotes Treg Cell Differentiation through Interaction with Foxp3

**Figure 5:**
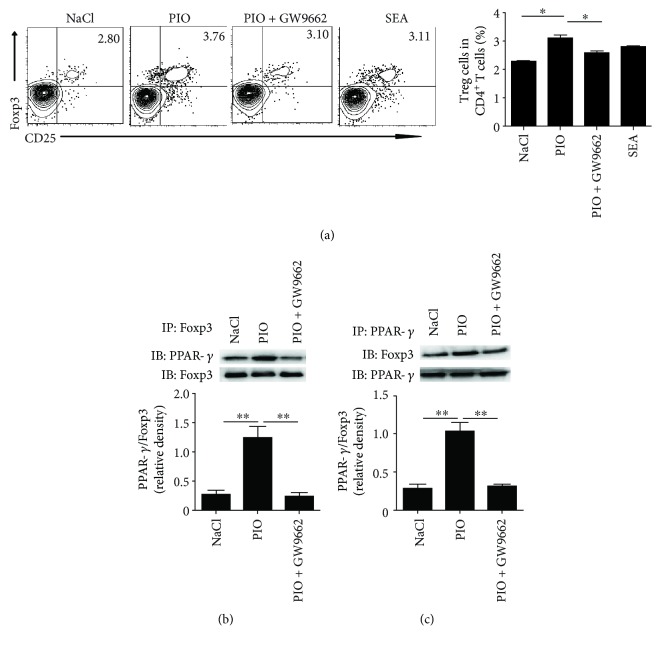
PPAR-*γ*/Foxp3 interaction promotes Treg differentiation. (a) CD4^+^ T cells (1 × 10^6^) were treated with pioglitazone (10 *μ*g/ml), SEA (25 *μ*g/ml), or (and) GW9662 (2 ng/ml) for 24 h, and then Treg (CD4^+^CD25^+^Foxp3^+^) cells were analyzed by FACS. (b) Normal purified CD4^+^ T cells of WT mice were stimulated with pioglitazone or GW9662 for 24 h, and then CD4^+^ T cell fractions were subjected to immunoprecipitation (IP) with an anti-PPAR-*γ* antibody, and the immunoprecipitates were analyzed by immunoblotting (IB) with anti-Foxp3 or anti-PPAR-*γ* antibody. (c) The association of PPAR-*γ* and Foxp3 was confirmed by a reciprocal immunoprecipitation assay using anti-Foxp3. All experiments were repeated three times with similar results (ANOVA/LSD), ^∗^*P* < 0.05 and ^∗∗^*P* < 0.01.

Considering that PPAR-*γ* was preferentially expressed in T cells [28], we next investigated whether pioglitazone could act on CD4^+^ T cells directly and promote Treg cell differentiation. Results showed that the percentage of Treg cells was significantly increased after direct pioglitazone stimulation (*P* < 0.01). However, the percentage of Treg cells was significantly decreased when the PPAR-*γ* inhibitor was used (*P* < 0.01) (Figure 5(a)). However, SEA-treated CD4^+^ T cells alone failed to cause an increased proportion of Treg cells, suggesting that SEA-induced CD4^+^ T cell differentiation required the presence of antigen-presenting cells. These results indicated that the activation of PPAR-*γ* by an agonist could directly promote differentiation of CD4^+^ T cells into Treg cells.

To determine whether PPAR-*γ* promoted CD4^+^ T cell differentiation through interaction with Foxp3, CD4^+^ T cells were prepared and stimulated with pioglitazone *in vitro*. Results showed that treatment with a PPAR-*γ* agonist (pioglitazone) resulted in enhanced coexpression of PPAR-*γ* and Foxp3 (Figure 5(b)), both of which are key transcription factors for Treg cells. On the other hand, treatment with a PPAR-*γ* inhibitor (GW9662) suppressed the interaction between PPAR-*γ* and Foxp3 in CD4^+^ T cells (Figure 5(c)), which is accompanied by decreasing Treg cells. Altogether, these results demonstrated that pioglitazone modulates PPAR-*γ* interaction with Foxp3 in CD4^+^ T cells, which subsequently induced the percentage of Treg cells and regulated the immune function.

## 4. Discussion

Although it has been well recognized that regulatory T cells play important roles in regulating immune response to infection, the mechanism involved in the regulatory function to schistosome infection has remained largely unclear. In this study, we for the first time report that PPAR-*γ* interacts with Foxp3 to promote the differentiation of CD4^+^ T cells into Treg cells and subsequently attenuating immunopathology during schistosome infection.

The main pathology in schistosomiasis results from eggs deposited in the liver, which impacts on the hosts' living quality, health status, or even mortality [5]. Interestingly, our results found that pioglitazone treatment could significantly improve the liver and spleen pathologies by HMI, spleen index, HE, and Masson staining. In addition, treatment with pioglitazone had little impact on egg production, while praziquantel reduced the number of eggs in the liver. These data suggested that pioglitazone played an important role in moderating hepatic and splenic pathologies but had no effect on the killing of adult worms during *S. japonicum* infection.

CD4^+^ T cells are the key factors involved in the development of immunopathology in schistosomiasis [5]; however, pioglitazone treatment did not influence the numbers of CD4^+^ T cells in *S. japonicum*-infected mice. Treg cells play a pivotal role in maintaining immune homeostasis [29, 30] and regulating the development of immunopathology in schistosomiasis [18, 19, 31]. Studies had showed that *Schistosoma* eggs can induce a marked Treg cell response, which prevents Th1 development and Th2 response [21]. Our results showed that pioglitazone treatment increased the expression of Treg cells, but decreased Th1/Th2 responses in *S. japonicum*-infected mice. Consistently, *in vitro* treatment of splenocytes with the PPAR-*γ* agonist and (or) SEA also induced the mRNA levels of Foxp3 and the percentage of Treg cells, while the PPAR-*γ* antagonist blocked this enhancement. Our results are supported by a study regarding inflammatory bowel disease (IBD) which used PPAR-*γ* deficient mice [32].

PPAR-*γ* can be expressed in macrophages [24, 25, 33], which are also the main cellular constituents of granulomas and important regulators in schistosomiasis [26, 27]. It is clear that PPAR-*γ* plays an important role in the regulation and maintenance of M2-type polarization [34–37]. Our results showed that PPAR-*γ* signaling is involved in the activation of macrophages and induction of CD206^+^ macrophages by using pioglitazone agonists and antagonists. In addition, we also found that activating PPAR-*γ* signaling in macrophages promoted Treg cell differentiation when cocultured with CD4^+^ T cells. This is supported by Savage et al.'s study, which indicated that human anti-inflammatory M2 macrophages induce Foxp3^+^GITR^+^CD25^+^ regulatory T cells [38].

To further clarify whether PPAR-*γ* activation could affect Treg cell differentiation directly, we used pioglitazone to stimulate purified spleen CD4^+^ T cells, and we found that PPAR-*γ* signaling could induce the expression of Foxp3 and increase the percentages of Treg cells. It has been reported that visceral adipose tissue- (VAT-) resident Tregs specifically express PPAR-*γ*, which appears to interact with Foxp3 in VAT-Treg cells. Indeed, ectopic coexpression of Foxp3 and PPAR-*γ* in conventional T cells induces a VAT-Treg-type gene-expression profile [22]. Our results showed that through interaction with Foxp3 in CD4^+^ T cells, PPAR-*γ* can induce the percentage of Treg cells and regulate immune response.

## 5. Conclusions

Our data showed that pioglitazone could induce CD4^+^CD25^+^Foxp3^+^ T cells by activating PPAR-*γ*/Foxp3 signaling, and subsequently suppressing the Th2-mediated immunopathology during *S. japonicum* infection. This study will deepen our understanding of the mechanism of the PPAR-*γ*/Foxp3 axis in the immunological effects of schistosome infection.

## Data Availability

The authors declare that the data supporting the findings of this study are available within the article, or from the authors on reasonable request.
